# Metabolomic analysis of serum short-chain fatty acid concentrations in a mouse of MPTP-induced Parkinson’s disease after dietary supplementation with branched-chain amino acids

**DOI:** 10.1515/med-2023-0849

**Published:** 2023-11-29

**Authors:** Na Mi, Lili Ma, Xueying Li, Jia Fu, Xinxin Bu, Fei Liu, Fan Yang, Yali Zhang, Lifen Yao

**Affiliations:** Department of Neurology, First Affiliated Hospital of Harbin Medical University, Harbin 150007, China; Department of Neurology, Chifeng Municipal Hospital, Inner Mongolia Autonomous Region, 024000, China; Department of Neurology, Chifeng Municipal Hospital, No. 1, Middle Section of Zhaowuda Road, Hongshan District, Chifeng City, Inner Mongolia Autonomous Region, 024000, China; Department of Neurology, First Affiliated Hospital of Harbin Medical University, No. 23, Youzheng Street, Nangang District, Harbin 150007, China

**Keywords:** metabolomic analysis, short-chain fatty acid, MPTP-induced, Parkinson’s disease, branched-chain amino acids

## Abstract

The gut microbiota and microbial metabolites influence the enteric nervous system and the central nervous system via the microbial–gut–brain axis. Increasing body of evidence suggests that disturbances in the metabolism of peripheral branched-chain amino acids (BCAAs) can contribute to the development of neurodegenerative diseases through neuroinflammatory signaling. Preliminary research has shown that longitudinal changes in serum amino acid levels in mouse models of Parkinson’s disease (PD) are negatively correlated with disease progression. Therefore, the aim of the present study was to determine the changes in serum levels of short-chain fatty acids (SCFAs) in a mouse model of 1-methyl-4-phenyl-1,2,3,6-tetrahydropyridine (MPTP)-induced PD after dietary BCAA supplementation. In our research, gas chromatography–mass spectrometry was used to detect serum SCFA concentrations. The data were then analyzed with principal component analysis and orthogonal partial least squares discriminant analysis. Finally, the correlations of serum SCFA levels with gut and motor function in MPTP-induced PD mice were explored. Propionic acid, acetic acid, butyric acid, and isobutyric acid concentrations were elevated in MPTP + H-BCAA mice compared with MPTP mice. Propionic acid concentration was increased the most, while the isovaleric acid concentration was decreased. Propionic acid concentration was positively correlated with fecal weight and water content and negatively correlated with the pole-climbing duration. In conclusion, these results not only suggest that propionic acid may be a potential biomarker for PD, but also indicate the possibility that PD may be treated by altering circulating levels of SCFA.

## 1 Introduction

Parkinson’s disease (PD) is the second most prevalent neurodegenerative disease; it is characterized by abnormal accumulation of α-synuclein (α-syn) and loss of dopaminergic neurons in the substantia nigra (SN) [1]. The motor symptoms of PD include bradykinesia, rigidity, tremor, and changes in gait. The nonmotor symptoms include functional decompensation, cognitive changes, upright hypotension, rapid eye movement sleep behavior disorder, and gastrointestinal issues such as constipation and delayed gastric emptying [2,3]. Furthermore, prior to the onset of motor symptoms in PD, pathological α-syn accumulation not only occurs in the SN, but throughout the enteric plexus as well as in the central nervous system (CNS). α-syn aggregates appeared in the dorsal motor nucleus of the vagus nerve in the early stages of the disease, with extensive connections to the enteric nervous system (ENS) [4]; thus researchers have suggested that the CNS pathology in PD may originate in the ENS [5]. This hypothesis emphasizes the contribution of alterations of the gut microbiota to the pathogenesis of PD [6,7]. Current treatments for PD have focused on delaying disease progression. However, further research into the pathogenesis of PD is needed to identify new therapeutic targets. Previous studies based on metabolomic approaches have shown that in PD mice model, disruption of branched-chain amino acid (BCAA) metabolism in the plasma correlates with the degree of disease progression [8]. Short-chain fatty acids (SCFAs), the metabolic end products of BCAAs, may exert protective effects on dopaminergic neurons. However, the specific mechanisms linking SCFAs and PD have not been fully elucidated. In the present study, we hoped to identify new therapeutic targets for the treatment of PD.

BCAA are essential amino acids such as leucine, isoleucine, and valine [9]. Gut microbes use BCAAs as substrates to produce SCFAs, including formic acid, acetic acid, propionic acid, isobutyric acid, butyric acid, isovaleric acid, and valeric acid. SCFAs are one of the major metabolites of the gut microbiota [10], and their production rate varies depending on the composition of the gut bacteria [10–12]. SCFAs can be used as a source of energy either directly by the colonic mucosa or indirectly via portal circulation [13] and exert neuroprotective effects by upregulating glial derived neurotrophic factors and brain-derived neurotrophic factors [14]. Accumulating data suggest that SCFAs have beneficial effects on regulation of brain function and blood–brain barrier integrity [15,16]. Additionally, SCFAs are required for normal microglial function in mice. Moreover, many studies have shown significantly lower SCFA concentrations in stool samples from human PD patients compared to those from healthy controls [7,17–19], and SCFAs are the primary regulators of accelerated neuroinflammation and α-syn in PD models [20].

Although numerous studies have investigated metabolomics in PD, few have examined how dietary supplementation with BCAAs alters serum SCFA levels to improve PD symptoms. In this study, we sought to detect and describe changes in serum SCFA levels in a mouse model of PD (established by treatment with 1-methyl-4-phenyl-1,2,3,6-tetrahydropyridine [MPTP]) following dietary BCAA supplementation. We employed gas chromatography–mass spectrometry (GC–MS) to identify putative PD biomarkers.

## 2 Materials and methods

### 2.1 Animals, diet, and model establishment

Male C57BL/6 mice weighing 20–25 g, age 6–8 weeks were acquired from Changzhou Cavens Laboratory Animal Co. Mice were given at least 1 week of acclimation at room temperature under a 12 h light/dark cycle. Water and food were freely available. The Institutional Animal Care and Use Committee of the First Affiliated Hospital of Harbin Medical University approved the experimental procedure, which were carried out in accordance with the National Institutes of Health’s Guide for the Care and Use of Laboratory Animals. The mice were randomly divided into three groups (*n* = 9), the control group fed with the typical diet (CON), MPTP-induced PD mice fed with the typical diet (MPTP), and MPTP-induced PD mice fed with a high BCAA diet (MPTP + H-BCAA). The BCAA dietary intervention was started 7 weeks before the intraperitoneal injection of MPTP was administered and continued for a total of 8 weeks (i.e., up to 1 week after the MPTP injection). A detailed description of the amino acids added to the diet is provided in Table 1.

**Table 1:** Quantity of amino acids in the two diets (typical and high in BCAA)

Compounds	Normal	High-BCAAs
Phenylalanine	0	4.4
l-Cystine	0	3.4
Isoleucine	0	16.8
Proline	0	10.3
Lysine	0	6.5
Methionine	3	2.3
Casein	200	100
Threonine	0	3.4
Tryptophan	0	1.1
Valine	0	20
Histidine	0	2.3
Alanine	0	2.3
Arginine	0	3.2
Aspartic acid	0	5.7
Glutamic acid	0	18.4
Glycine	0	1.6
Leucine	0	31
Serine	0	4.8
Tyrosine	0	4.7
Total	203	241.9

### 2.2 Assessment of colonic function: 1 h stool collection

In the three groups of mice, the frequency of feces was tracked for a full hour. For this assessment, each mouse was placed in a separate, clean plastic cage without food or water. Following defecation, mouse feces were promptly collected in an individual, sealed 1.5 mL tube. To determine the wet weight of the feces, the tubes were weighed after collection. To obtain the dry weight of the feces, the tubes were reweighed after drying overnight at 65°C. The difference between dry and wet weight was used to compute the fecal water content.

### 2.3 Behavioral test: the pole-climbing test

Mice were placed head down on top of a pole (50 cm high, 1 cm in diameter, with a rough surface and a spherical projection at the top 2 cm in diameter). The total time that each mouse took to climb from the top to the bottom of the pole (i.e., climbing duration) was measured. Three repetitions of the pole-climbing test were performed for each animal, and statistical analysis was performed based on the average climbing duration.

### 2.4 Serum sample collection for metabolomics analysis

Mice were sedated with intraperitoneal injections of ketamine and thiazide (120 and 16 mg/kg, respectively), and blood was collected by removing the eyeball. Blood (1.5 mL) was placed in a coagulation-promoting tube and centrifuged at 3,000*g* for 30 min at 4°C to obtain the serum samples, which were stored at −80°C. These serum samples were used for metabolomic analysis by sequential GC‒MS. Finally, mice were sacrificed by cervical dislocation.

### 2.5 Metabolite sample preparation

After being prepared at −80°C, samples were defrosted at ambient temperature. For the experiment, 100 L of sample was mixed with 20 L of succinic acid (25 mol/L) and 600 L of a solution containing methanol:acetonitrile (2:1, vol/vol) and 0.1% formic acid. After sonication for 10 min, the mixture was placed at −20°C for 30 min and centrifuged at 13,900*g* for 10 min at room temperature. All samples were mixed to create quality control (QC) samples (one mixed sample). Supernatant (100 µL) was transferred to a glass sampling vial and dried under vacuum at room temperature; subsequently, 80 μL of methoxylamine hydrochloride (15 mg/mL) dissolved in anhydrous pyridine was added. The prepared mixture was vortexed vigorously for 2 min before being incubated at 37°C for 90 min. The sample was then treated with 50 L of N,O-bis(trimethylsilyl)trifluoroacetamide (containing 1% trimethylchlorosilane) and 20 L of hexane. The samples were vortexed vigorously for 2 min before being incubated at 70°C for 60 min. Finally, the samples were left at room temperature for 30 min before GC‒MS metabolomics analysis.

### 2.6 GC–MS analysis parameters

A Trace 1310/TSQ 9000 mass spectrometer system (Thermo, CA, USA) was used for GC–MS (Thermo, CA, USA). GC was carried out in a DB-5MS capillary column (30 m × 0.25 mm × 0.25 μm, Agilent J&W Scientific, Folsom, CA, USA). A steady flow of 1.2 mL/min helium was injected, with a purity limit of 99.999%. With the solvent delay time set to 4 min, a sample volume of 1 L was injected in splitless mode. The column incubator’s initial temperature was set to 50°C for 0.5 min. Next, the temperature was increased to 125°C at a rate of 15 °C/min for 2 min, then to 210°C at a rate of 8 °C/min for 2 min, 270°C at a rate of 11°C/min, and 305°C at a rate of 25°C/min; finally, 305°C was maintained for 3 min. Utilizing a selective reaction monitor with a mass range set to *m*/*z* 40–600, mass spectrometry data were collected. QC samples were examined on a regular basis (every ten samples).

### 2.7 Statistical analysis

Statistical analysis was performed using GraphPad Prism8 software. Data are expressed as the mean ± standard deviation. Behavioral data from the three groups of mice were analyzed by one-way analysis of variance (ANOVA).

#### 2.7.1 Metabolomics data analysis

The SIMCA 14.1 software package and MetaboAnalyst 5.0 were used for multivariate analysis including principal component analysis, orthogonal partial least squares (OPLS), and OPLS discriminant analysis (OPLS-DA). *R* ^2^ and *Q* ^2^ were calculated to assess the performance of the OPLS model. The model was then validated using a 200-iteration permutation test, and the model’s statistical significance was determined using a cross-validation ANOVA. To screen potential biomarkers, the variable importance of projection (VIP) of OPLS-DA was used. Finally, Spearman’s correlation analysis was used to assess the relationships between serum SCFA concentrations and mouse behavior. *P* < 0.05 was considered statistically significant.


**Ethical approval and consent to participate:** This study was approved by the ethics committee of the First Affiliated Hospital of Harbin Medical University. All animal experiment procedures were performed in accordance with the Guidance Suggestions for the Care and Use of Laboratory Animals, formulated by the Ministry of Science and Technology of China.

## 3 Results

### 3.1 Dietary BCAA supplementation improves gut and motor dysfunction in MPTP-induced PD mice

**Figure 1:**
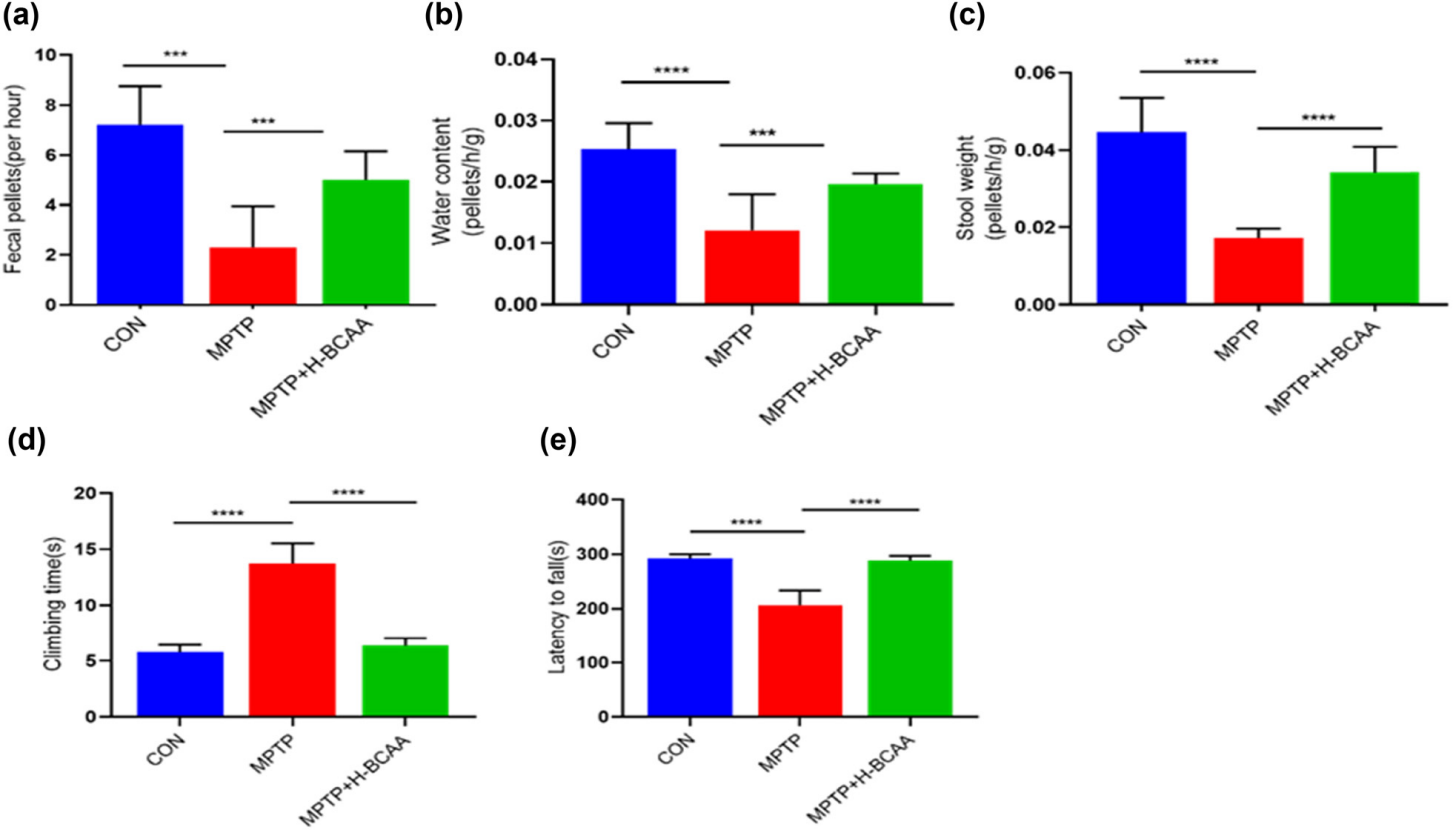
Dietary BCAA supplementation reduces bowel and motor dysfunction: (a) fecal excretion volume, (b) fecal water content, (c) fecal weight, (d) climbing duration in the pole-climbing test, and (e) latency to fall from the bar in the rotating bar test. *, *P* < 0.05; **, *P* < 0.01; ***, *P* < 0.001.

The effect of the H-BCAA diet on MPTP-induced intestinal dysfunction was investigated by observing mouse fecal excretion over 1 h. As shown in Figure 1a–c, the 1 h defecation frequency, fecal weight, and fecal water content of the MPTP group were significantly lower than those of the CON and MPTP + H-BCAA groups; however, there were no significant differences in the 1 h defecation frequency, fecal weight, and fecal water content of the MPTP + H-BCAA and CON groups. In the pole-climbing test, the climbing duration of mice in the MPTP group was significantly longer than that of mice in the CON and MPTP + H-BCAA groups; there was no significant difference in the climbing duration of mice in the MPTP + H-BCAA and CON groups (Figure 1d). In the rotating bar test, the duration that mice in the MPTP group stayed on the rotating bar (i.e., the latency to fall) was significantly shorter than that of mice in the CON and MPTP + H-BCAA groups; there was no significant difference in the latency to fall of mice in the MPTP + H-BCAA and CON groups (Figure 1e). These findings suggest that the H-BCAA diet effectively improved MPTP-induced intestinal and motor dysfunction in mice.

### 3.2 Metabolomic characterization of the three groups

**Figure 2:**
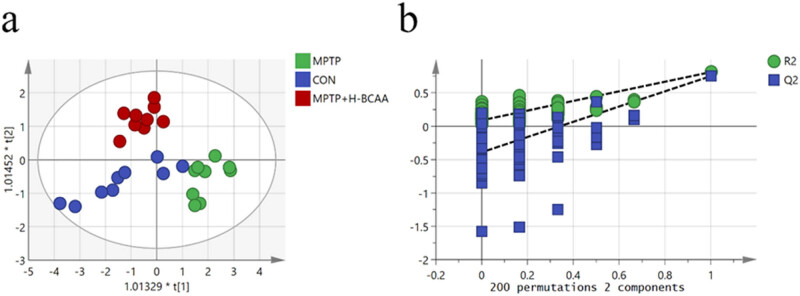
Supervised multivariate data analysis of mouse metabolites. (a) Plots of supervised OPLS-DA scores for SCFA concentrations in the MPTP, CON, and MPTP + H-BCAA groups. *R* ^2^_*X*_ = 0.804, *R* ^2^_*Y*_ = 0.736, *Q* ^2^ = 0.627. (b) Results of 200 iterations of permutation test.

**Figure 3:**
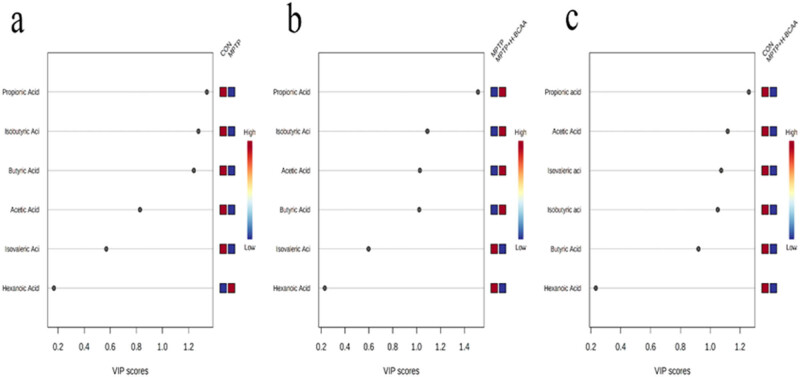
VIP scores of the OPLS-DA model for screening differential metabolites: (a) VIP scores of the CON and MPTP groups, (b) VIP scores of the MPTP and MPTP + H-BCAA groups, and (c) VIP scores of the CON and MPTP + H-BCAA groups.

To further clarify the changes in serum SCFA levels of PD mice after dietary BCAA supplementation, serum samples were analyzed by a GC‒MS-based targeted metabolomics method. Serum levels of six SCFAs (acetic acid, butyric acid, caprylic acid, isobutyric acid, isovaleric acid, and propionic acid) were measured. An OPLS-DA model was constructed to further clarify the effect of BCAA supplementation on SCFA metabolism. A significant separation of SCFA levels among the groups was observed (*R* ^2^_*X*_ = 0.804, *R* ^2^_*Y*_ = 0.736, *Q* ^2^ = 0.627) (Figure 2a). The predictive ability of the OPLS-DA model was evaluated with a substitution test with 200 iterations (Figure 2b). Differences in SCFA concentrations between the CON and MPTP groups (Figure 3a), the MPTP and MPTP + H-BCAA groups (Figure 3b), and the CON and MPTP + H-BCAA groups (Figure 3c) were identified by the OPLS-DA model. We found that the VIP scores of propionic acid, isobutyric acid, and butyric acid were greater than 1 in the CON and MPTP comparison, than those of propionic acid, isobutyric acid, butyric acid, and acetic acid which were greater than 1 in the MPTP and MPTP + H-BCAA comparison, and that of propionic acid, acetic acid, isobutyric acid, and isovaleric acid were greater than 1 in the CON and MPTP + H-BCAA comparison.

### 3.3 Group differences in SCFA concentrations

A heatmap was used to identify the differences in SCFA concentrations among the three groups. The concentrations of propionic acid, butyric acid, isobutyric acid, and acetic acid were lower in the MPTP group than in the MPTP + H-BCAA group, while the concentration of isovaleric acid was higher in the CON and MPTP groups than in the MPTP + H-BCAA group. The concentrations of butyric acid, isobutyric acid, and acetic acid were higher in the MPTP + H-BCAA group than in the MPTP group, but this difference was not significant.

**Figure 4:**
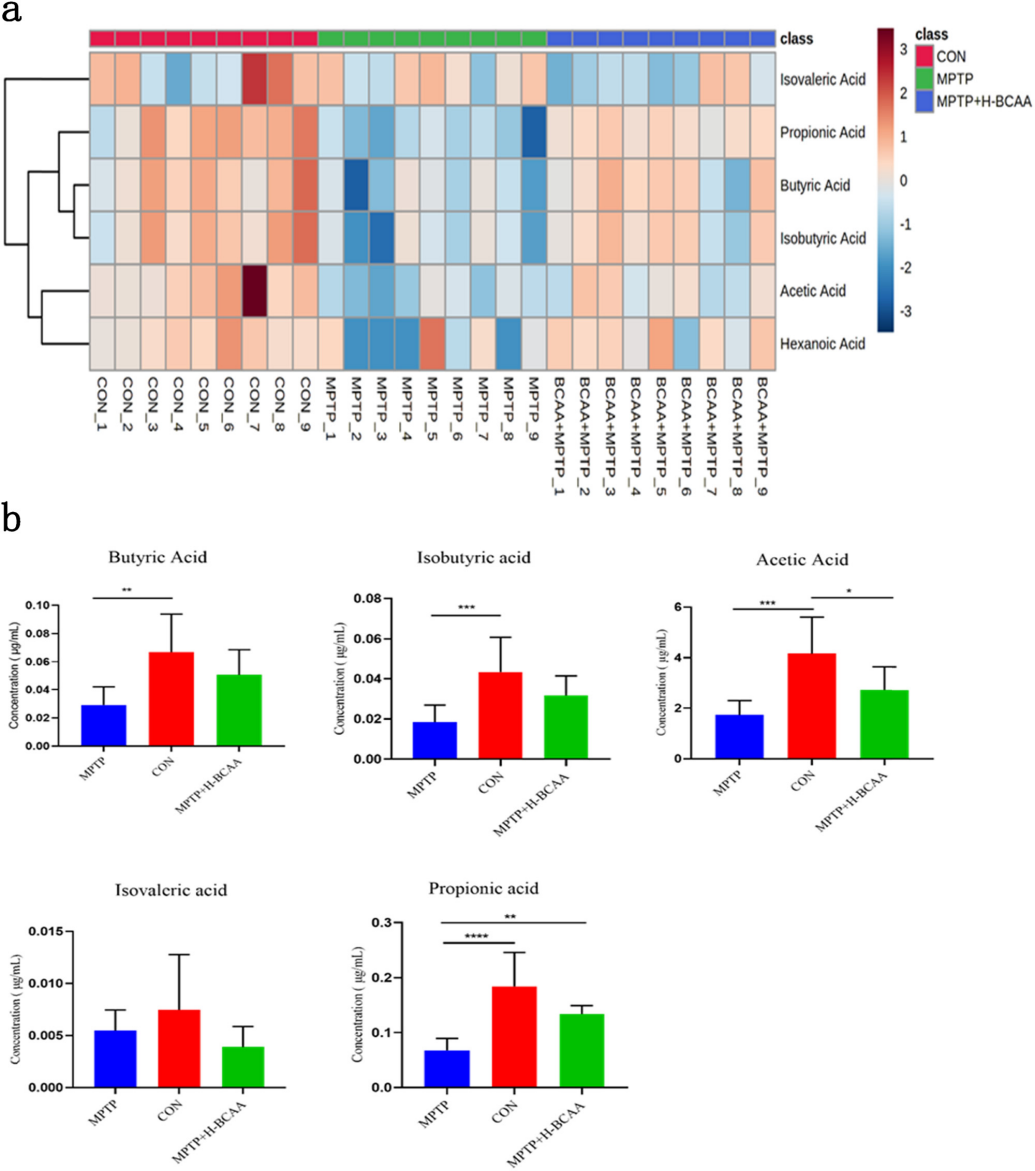
(a) Relative levels of SCFA in each group of mice displayed in a heatmap. The degree of change is indicated by the color. (b) Box plots showing that the propionate level in the MPTP group was significantly lower than that in the MPTP + H-BCAA group. *, *P* < 0.05; **, *P* < 0.01; ***, *P* < 0.001.

Although the differences in acetic acid, butyric acid, and isobutyric acid concentrations between the MPTP + H-BCAA group and the CON group were not statistically significant, the concentrations of these three SCFAs showed an increasing trend in the MPTP + H-BCAA group compared with the MPTP group. Among these SCFAs, only propionic acid was significantly higher in the MPTP + H-BCAA group than in the MPTP group (*P* < 0.01) (Figure 4b).

**Figure 5:**
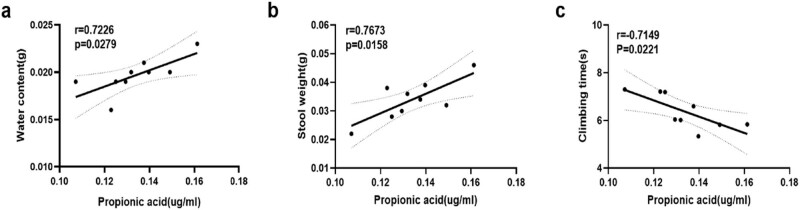
Correlations of gut and motor function with propionic acid concentrations in the MPTP + H-BCAA group: (a) correlation of propionic acid concentration and fecal water content (*r* = 0.7226, *P* = 0.00279), (b) correlation of propionic acid concentration and fecal weight (*r* = 0.7673, *P* = 0.0158), and (c) correlation of propionic acid concentration and climbing duration (*r* = 0.44, *P* > 0.05).

Finally, we performed a correlation analysis to determine whether the propionic acid concentration correlated with gut and motor function in the MPTP + H-BCAA group. The propionic acid concentration was negatively correlated with the pole-climbing duration of mice and positively correlated with the fecal water content and fecal weight (Figure 5).

## 4 Discussion

We found that dietary BCAA supplementation improved both motor and nonmotor symptoms of PD and promoted SCFA production in a mouse model of PD. According to previous reports, BCAA supplementation prolonged mouse lifespan [21], improved cognitive function in a mouse model of Alzheimer’s disease [22], reduced mouse body weight [23], and increased adiponectin secretion and insulin sensitivity [24,25]. This study shows that dietary BCAA supplementation improves SCFA levels as well as motor and nonmotor symptoms in PD mice.

The OPLS-DA model revealed a significant separation of the three groups in terms of SCFA levels. Some researchers have suggested that changes in SCFA concentrations may be related to dysregulation of the gut microbiome. PD patients exhibit different levels of intestinal inflammation [26], reflected in the increase in pro-inflammatory Enterobacteriaceae [6,17,27], disruption of the intestinal barrier [28], and elevation of PD risk in inflammatory bowel disease patients. Taken together, these results suggest that in PD patients, the intestinal barrier is disrupted and that SCFAs in the intestine may enter circulation through the damaged intestinal barrier; thus, plasma SCFA levels indirectly reflect the SCFA levels in the intestine. Dysbiosis of the gut microbiota and reduced SCFA concentrations in stool have been reported in PD patients [17]. In addition, SCFA-producing bacteria [29], especially Trichophytonaceae [30], have been observed at low levels in stool samples from PD patients. An MPTP-induced mouse model of PD exhibited a similar dysbiosis of the gut microbiota [31]; as well as a decrease in SCFA-producing bacteria, such as the taxa Phyllobacterium and Thickobacteria [32]. The addition of BCAAs increased the levels of Phyllobacteriaceae, Trichosporonaceae, and Ruminococcaceae in the intestine [33], which further suggests that BCAA supplementation in PD mice may have altered the composition of the gut microbiome, which in turn increased SCFA concentrations.

To further elucidate the effects of SCFA levels on individuals, we examined the changes in serum SCFA levels using targeted metabolomics via GC‒MS. Compared to the MPTP group, the MPTP + H-BCAA group had higher concentrations of propionic acid (*P* < 0.01), butyric acid, isobutyric acid, and acetic acid but a lower concentration of isovaleric acid. The propionic acid concentration was correlated with motor and nonmotor symptoms in the MPTP + H-BCAA group. Thus, acetic acid, butyric acid, isobutyric acid, and propionic acid play an important role in the pathogenesis of PD. As the propionic acid concentration showed the most significant difference and was correlated with motor and nonmotor symptoms, we suggest that it may be a promising intervention target in PD. Some studies have shown that propionic acid is negatively correlated with score on the Unified Parkinson’s Disease Rating Scale part III [17], suggesting that propionic acid may be beneficial in PD.

Studies have documented numerous advantages of propionate for physiological processes; for example, it attenuates the lipopolysaccharide-induced epithelial–mesenchymal transition [34], encourages intestinal gluconeogenesis [35], modifies the progression of multiple sclerosis by reversing Treg cell/Th17 cell induction, and improves Treg cell function [36]. Propionate can easily cross the blood–brain barrier; in fact, GC studies have shown a high concentration of propionate in the hippocampus [37]. Other research has demonstrated that propionate exerts these effects by promoting the release of glucagon-like peptide 1 (Glp-1) from enteroendocrine L cells or by prompting the peripheral nervous system to send signals to the brain. Glp-1 provides clinical advantages in the treatment of moderate PD [38,39]. Alvare et al. also demonstrated that expression of GLP-1 receptor (GLP-1R) occurs in the SN [40] and that microglia respond to inflammatory stimuli by increasing the expression of GLP-1 and GLP-1R [41], which also suggests that propionate may exert a protective effect on dopaminergic neurons by suppressing the nigrostriatal inflammatory response. We showed that propionic acid improves motor and nonmotor symptoms in PD mice, which is likely based on the above mechanism of action.

There are several limitations to our study. First, our study primarily demonstrated the protective effects of SCFAs, especially propionate, rather than the therapeutic effect. In future studies, we plan to further explore the therapeutic effects of propionate administration after successful establishment of PD models. Second, it remains unclear which signaling pathways are influenced by propionate; we plan to investigate this in the future. In conclusion, our findings provide a new avenue for PD treatment (i.e., dietary intervention) by changing the metabolites of the gut microbiota of PD mice and improving motor and nonmotor symptoms. We hope that in the future, therapy for PD will be possible not only through drugs but also through dietary interventions to improve PD symptoms and provide more possibilities for the treatment of PD patients.
